# Postjunctional M2 Muscarinic Receptors Augment Neurally Mediated Cholinergic Contractions of Murine Airway Smooth Muscle

**DOI:** 10.1093/function/zqab067

**Published:** 2021-12-23

**Authors:** Sean M Ward

**Affiliations:** Department of Physiology and Cell Biology, University of Nevada, Reno School of Medicine, Reno, NV 89557, USA

**Keywords:** airway smooth muscle, cholinergic contraction, muscarinic receptors, asthma, chronic obstructive pulmonary disease

A Perspective on “Contribution of postjunctional M2 muscarinic receptors to cholinergic nerve-mediated contractions of murine airway smooth muscle”.

Airway bronchoconstriction is primarily controlled by the vagus that releases acetylcholine (ACh) and activates post-junctional muscarinic ACh receptors (mAChRs) on airway smooth muscle (ASM). These parasympathetic nerves tonically active at rest, produce a stable tone of ASM.^1^ The major populations of mAChRs located post-junctionally on ASM are of the M2 and M3 subclasses. Contraction of ASM is thought to be mediated primarily through activation of G_q_-coupled M3AChRs. Stimulation of M3AChRs leads to activation of phospholipase C and its cleavage of phosphatidylinositol 4,5 biphosphase (PIP2) into diacylglycerol (DAG) and inositol 1,4,5-trisphosphate (IP3). IP3 causes release and subsequent rise in intracellular calcium in ASM leading to ASM contraction. M2AChRs, which couple to the G-protein G_αo/i_, are highly expressed on ASM and their activation via G_s_, inhibits adenylate cyclase with subsequent reduction in cyclic adenosine monophosphate (cAMP) generation and it's accumulation.^2^ Although postjunctional ASM M2AChRs outnumber M3AChRs in a variety of species by a ratio of up to 4:1, their role in cholinergic ASM contractions is thought to be negligible.^3^ M3AChRs are thought to mediate the bronchoconstrictor effects of acetylcholine rather than M2AChRs. M2AChRs are also located on pre-junctional membranes of nerve fibers within the airways where they are thought to regulate negative feedback reducing ACh release from parasympathetic nerve terminals.^4^ Since pre-junctional M2AChRs act as negative feedback regulators, their inhibition would have an adverse effect for the treatment of asthma or COPD, as this would tend to enhance ACh release and cause further bronchoconstriction. It has previously been demonstrated that in small airways of M3AChR null mice a significant cholinergic contractile response persisted, and this was abolished in mice with both M3 and M2AChRs knocked out.^5^ These data provide evidence that both mAChR subtypes are involved in cholinergic contractile responses in ASM. mAChRs have been targets for the treatment of asthma and chronic obstructive pulmonary disease (COPD). Studies have demonstrated the potential beneficial effects of the use of mAChR antagonists in the treatment of these disorders and are thus primary therapeutic targets for these conditions.^2^

An article published in this issue of *Function* from the Smooth Muscle Research Center at the Dundalk Institute of Technology and the School of Medicine, Queens University, Belfast, Ireland,^6^ describes a possible explanation to a formidable contribution of post-junctional M2AChRs on ASM contractions. Using low-frequency short-duration pulses of electric field stimulation (EFS) to excite intramural parasympathetic nerves, the authors demonstrate M2AChR-mediated hypersensitization of M3AChR-dependent contractions of ASM that markedly augmented EFS evoked contractions. Contractions evoked by stimulus frequencies of 2 Hz and pulse durations of 0.3 ms, to selectively activate intramural nerve fibers, were increased >2.5-fold when the stimulus interval was reduced from 100 to 10 seconds. The augmentation in EFS evoked contractions with 10 second stimulus intervals was reversed with M2AChR antagonists and was absent in M2-null mice, thus providing definitive evidence of the role of M2AChRs in this enhanced contractile response. Further, a M3AChR antagonist abolished the augmentation, providing a link between M2 and M3AChRs in the potentiated contractions.

M2AChRs are G_i_-protein-coupled receptors that upon their activation in visceral smooth muscles reduce intracellular cAMP by inhibiting adenylate cyclase,^7^ and have been implicated in cholinergic-mediated contractile responses in several visceral organs. However, as stated above their role in cholinergic ASM contractions is thought to be unimportant. It has been reported that rather than contribute directly to contractions they offer a brake on the inhibitory actions of β-adrenoceptor (β-AR) activation that leads to a rise in intracellular cAMP and subsequent relaxation of ASM.^7^

In the study by Alkawadri *et al.*,^6^ it is clearly demonstrated that EFS evoked cholinergic contractions elicited by low frequency, i.e. 2 Hz at 100s intervals (Figure 1) produced modest contractions that were unaffected by M2AChR antagonists but were abolished by a M3AChR antagonist. Using the same parameters but decreasing the interval from 100s to 10s revealed a M2AChR component that increased the contractile response by >250%, was blocked by M2AChR antagonists and was absent in M2AChR null mice (Figure 1).

**Figure 1.**
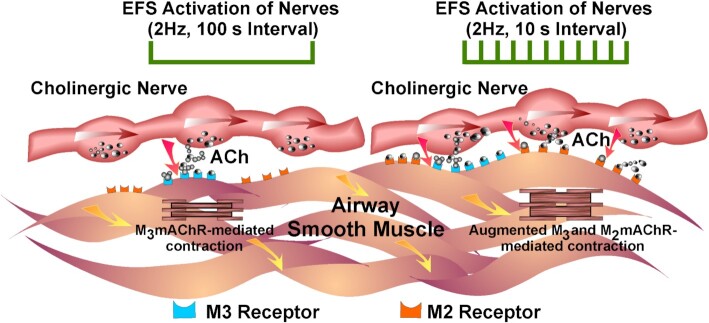
Model for contribution of M2AChR augmentation of cholinergic mediated contractions of airway smooth muscle. Electrical field stimulation (EFS) using short duration pulses (0.3 ms) at a low stimulus frequency (2Hz) delivered at intervals of 100 seconds to selectively stimulate intramural nerves evoked contractions that were mediated by M3AChRs (left hand side of figure). Shortening the stimulus interval from 100 to 10 seconds produced a marked sensitization of the cholinergic contractile response (augmenting the response >2.5 fold). This augmentation involved the recruitment of M2AChRs (right hand side of figure).

As with any outstanding study, new questions arise, and further experiments are needed to fully explain the mechanism(s) underlying the activation of M2AChRs. The cholinergic contractile response was inhibited by tetrodotoxin, providing evidence that it was due to action potential-dependent mechanisms in nerve fibers and not ACh release from other cellular phenotypes in ASM that have the capability to synthesize ACh.^8^ The logical thought is that it would be overflow of ACh onto extra-junctional M2AChRs. This is supported by the observation that inhibition of acetylcholinesterase (AChE) induced contractions in ASM were blocked by the M2AChR antagonist, methoctramine. However, AChE hydrolyzes choline esters with an extremely high catalytic rate, each molecule degrading approximately 25 000 molecules per second, approaching the diffusion rate of the substrate, and making it one of the most efficient enzymes known.^9^ Thus, although the data supports the activation of extrajunctional M2AChRs, the presence of AChE in the extracellular space between nerve terminals and ASM cells would likely be sufficient in hydrolyzing any ACh overflow. It is possible that nerve terminals and post-junctional neuroeffector cells in ASM are like that reported for the stomach, where synaptic specializations exist between nerve terminals and specialized interstitial cells of Cajal (ICC). In the stomach EFS evokes anoctamin-1-dependent cholinergic excitatory junction potentials that are absent when intramuscular ICC are deficient. In the presence of AChE EFS evokes a slower developing cholinergic excitation likely due to overflow of ACh onto non-junctional M2AChRs that activate a non-selective cation conductance expressed on smooth muscle cells.^10^ Further studies performed by the authors will hopefully resolve these questions in ASM.

In summary, Alkawadri *et al*. ^6^ presents a highly novel role for post-junctional M2AChRs that provide a sensitization mechanism of M3AChR nerve mediated cholinergic responses in ASM. The role of M2AChRs in potentiating neurally mediated cholinergic post-junctional responses opens new avenues to pursue these receptors with specific antagonists for the treatments of ASM diseases such as asthma and COPD, if a balance between pre-junctional and post-junctional M2 responses can be discriminated.

## Funding

The work was supported by the National Institutes of Health (grant NIH DK057236).
